# Unusual implant-related soft tissue reaction presenting as a swollen leg: a case report

**DOI:** 10.1186/1752-1947-8-187

**Published:** 2014-06-13

**Authors:** Seyma Ozkanli, Mehmet Salih Soylemez, Adem Sahin, Serkan Senol, Kerem Bilsel, Hasan Hüseyin Ceylan

**Affiliations:** 1 Department of Pathology, Göztepe Training and Research Hospital, Fahrettin Kerim Gökay Street, 2346 Istanbul, Turkey; 2 Department of Orthopedics and Traumatology, Göztepe Training and Research Hospital, Fahrettin Kerim Gökay Street, 2346 Istanbul, Turkey; 3 Department of Orthopedics and Traumatology, School of Medicine, Bezmialem Vakıf University, Vatan Street, 34080 Istanbul, Turkey; 4 Göztepe E.A.H. floor 3. Orthopaedics ward, Fahrettin Kerim Gökay Street, 2346 Kadıköy/Istanbul, Turkey

**Keywords:** Soft-tissue reaction, Granuloma, Malignancy, Orthopedic hardware

## Abstract

**Introduction:**

There are several causes of peri-implant edema, pain, and swelling around implants after orthopedic fixation device application for fracture repair. The most common and well-known reason is infection, however, granulomas associated with foreign body reactions are rarely seen. In this report we present a case of a granulomatous reaction mimicking a hydatid cyst and sarcoma. We emphasize the importance of differential diagnosis in triggering appropriate management of the patient. Our case was unusual; to the best of our knowledge no similar clinical or pathological findings have been reported in current literature.

**Case presentation:**

A 56-year-old Turkish man who had been treated for a right femoral fracture via a plate fixation 10 years prior underwent an operation to treat pain and swelling around the plate. A hydatid cyst-like mass was observed during surgery, but subsequent examination revealed that no hydatid cyst was present; both malignancy and infection were also absent.

**Conclusions:**

Although infection is generally the first possibility that should be considered in a patient complaining of pain and swelling in the vicinity of an implant, malignancies, hydatid cysts, and (finally) foreign body reactions should also be kept in mind as differential diagnoses. A soft-tissue reaction around a previously implanted plate should be managed carefully. Pre-operative radiological assessment, and biopsy to allow pathological and microbiological examination, should be considered in all suspected cases.

## Introduction

There can be several causes of peri-implant edema, pain, and swelling around the implant after orthopedic fixation device application for fracture repair. The most common and well-known reason is infection however, granulomas associated with foreign body reactions are rare. Swelling and pain may develop around the implant in patients with sarcomas. *Staphylococcus* spp. are the most common infectious organisms associated with orthopedic implant surgery. Furthermore, hydatid cysts, especially in patients with predisposing factors, may cause implant infections [1]. *Echinococcus granulosus* is usually responsible for the more commonly encountered hydatid diseases and/or infections [2]. Although uncommon, the development of a sarcoma due to the presence of an orthopedic implant can be caused by swelling after the implantation. To the best of our knowledge, thirty-one cases of sarcoma development due to an orthopedic implant have been reported in current literature [3].

Sarcoma development due to implants can take several forms, but the most common are malignant fibrous histiocytoma and osteosarcoma. In addition, there has been one report of a peripheral nerve-sheath tumor and two reports of lymphoma resulting from implants [3]. These types of tumoral mass behave aggressively and frequently metastasize. Clinically, these cases should be distinguished from non-neoplastic reactions, such as infection, and reactions to the prosthetic debris associated with implants. Our case report involves a patient who had been treated for a right femoral fracture by fixation with a plate. Ten years later the patient underwent an operation due to pain and swelling around the plate. During surgery, a hydatid cyst-like mass was observed, but subsequent examination revealed no malignancy, infection, or hydatid cyst.

## Case presentation

**Figure 1:**
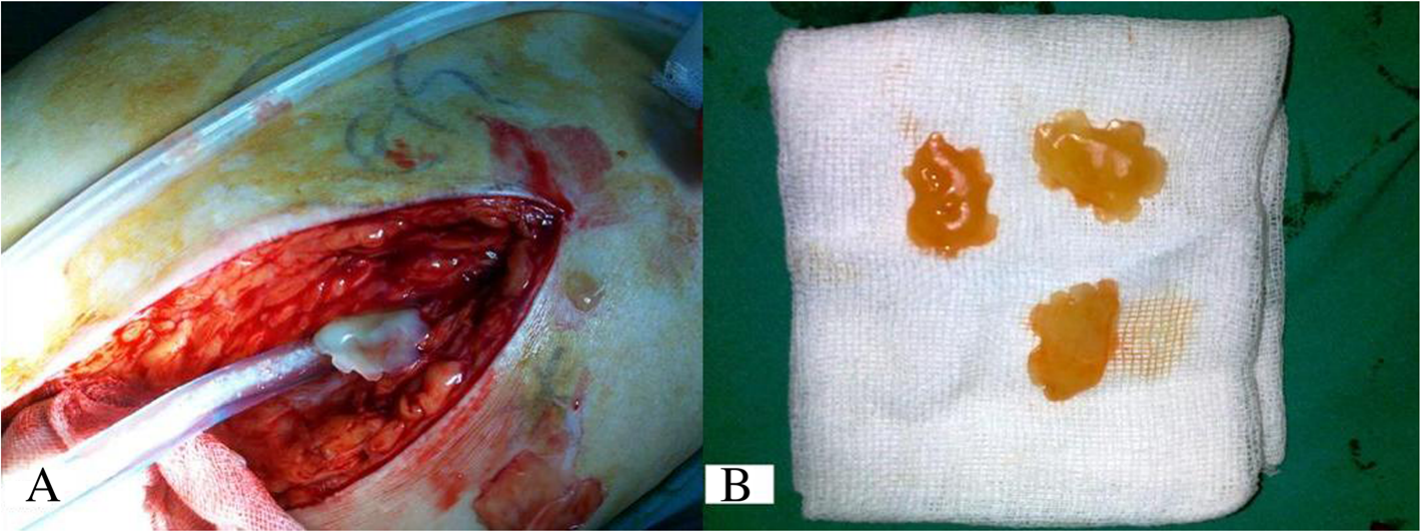
Image of snow-shaped vesicles extracted from the mass (A, B).

**Figure 2:**
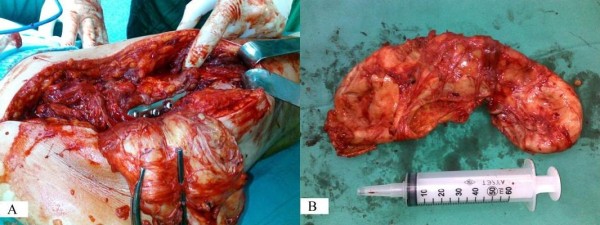
Image of the capsule, dissected from the stainless steel plate (A, B).

A 56-year-old Turkish man with no history of illness or drug use was treated with plate and screw osteosynthesis because of a right femoral fracture after a vehicle accident in 1998. He had been living with and feeding a dog for the past 16 years. Two years prior to the current presentation, he noticed a slight swelling on the proximal lateral aspect of his right thigh, but he did not pursue evaluation. However, the symptoms had been increasing during the most recent six months, and he was admitted to our clinic. His C-reactive protein (CRP) level was 3.7mg/dl and his eosinophil concentration was 12.4%. A biopsy specimen was taken from the area of swelling because of suspected infection. The biopsy specimen was considered to involve greater trochanter bursitis. However, because of increasing swelling and pain during the three months following the biopsy, he was re-evaluated in our clinic. A 25×12cm mass was present on the proximal anterolateral aspect of the right thigh, starting at the greater trochanter and spreading both distally and posteriorly. There was no warmth or redness associated with the mass. A diagnosis of bursitis of the greater trochanter was made due to the swelling, which was particularly severe on the greater trochanter and the screw heads on the proximal greater trochanter. To identify the causative organism, the mass was punctured again. The culture grew methicillin-sensitive, coagulase-negative *Staphylococcus (Staphylococcus epidermidis)*, which was determined to be due to contamination. Tests for a potential infection were taken. His test results showed a sedimentation rate of 107mm/h, CRP of 1.38mg/dl, a white blood cell count of 7.8×10^3^/mm^3^, and eosinophils of 2.4. He was diagnosed with bursitis of the trochanter major, and removal of the bursitis and an implant operation were planned. The excised mass started from the lateral aspect of the distal femur (next to the implant) and spread postero-superiorly to the level of the greater trochanter, which was 15×12×6cm in diameter. The mass was filled with a 1×1cm cystic structure that appeared to be comprised of hydatid cyst vesicles (Figures 1 and 2). During surgery, the mass was excised with preservation of the sciatic nerve as the cyst had burst. Bone tissue had not developed on the implant and the plates and screws were removed from the patient. Intra-operatively, we considered that the mass might have been a hydatid cyst or a sarcoma, and thus post-operatively, the patient underwent contrast-enhanced abdominal and thoracic computed tomography (CT). His CT scan revealed neither a primary focus nor any other area of involvement. An abdominal ultrasonography examination was unremarkable. A direct parasitic examination of the specimens taken intra-operatively showed no protoscoleces within the cyst. Samples were taken intra-operatively and sent for pathological examination. The diagnosis was verified and the soft-tissue reaction evaluated. Twelve months after the procedure our patient was fully mobilized and determined to be in a good general condition. A follow-up CT scan of his abdomen, chest, and right thigh revealed no secondary focus.

## Discussion

Although implants are biologically inactive materials, they may cause soft-tissue reactions [4]. The most common causes of peri-implant swelling and pain in the mid- to long-term period after orthopedic internal fixation device implantation are infections and soft-tissue reactions, caused by metallic debris originating from these materials. *Staphylococcus* spp. are the most common causative organisms of infections that develop after orthopedic implant surgery [1]. In a study by Arciola *et al*. [1], *Staphylococcus* spp. were detected in 43 out of 50 (86%) orthopedic implants. Moreover, *Staphylococcus aureus* was the only detected microorganism in 34 patients (68%). In our case, the pre-operative radiographs were normal, there were no signs of a loosening of the implant, and the bone plate attachment was quite firm. The intra-operative cultures were sterile. The large number of snowflake-like cysts, 1×1cm in size and demonstrating a cystic structure, were thought to be hydatid cysts as our patient was known to have a dog.

The hydatid cyst, which contained an outer fibrous macroscopic laminar layer and an inner germinal layer, had a so-called two-layered cyst wall containing a clear liquid. The germinal membrane contained small cysts and protoscoleces. Hydatid-related skin [5], brain [6], pancreas [7], leg, and spine [8] disease has been reported, but such a disease would not be expected to cause orthopedic implant infections. Therefore, local changes in the incision after a previous orthopedic surgery may direct the practitioner to investigate other bacterial pathogens, potentially leading to diagnostic errors. If a patient’s laboratory results are atypical at the wound site of a non-specific infection following orthopedic surgery, and the patient has a history of travelling to a region endemic for hydatid disease as well as contact with dogs, a diagnosis of hydatid cyst disease should be considered [9]. However, in our patient, his post-operative tests revealed no evidence of hydatid disease. The intra-operative findings were indicative of hydatid disease but, upon pathological and parasitological examination, neither scolices nor protoscoleces were detected.

**Figure 3:**
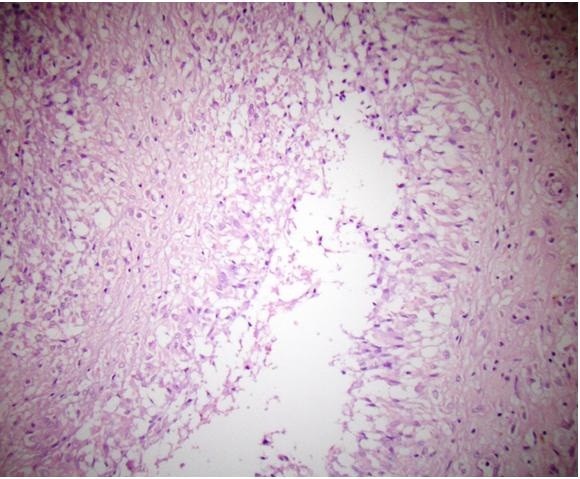
**Lymphocytic and histiocytic inflammation in soft tissue.** (Stain, hematoxylin and eosin; original magnification, ×100).

**Figure 4:**
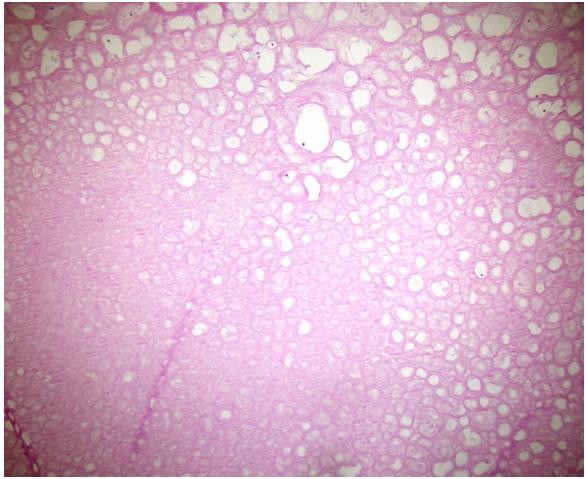
**Acellular fibro-hyalinized structure.** (Stain, hematoxylin and eosin; original magnification, ×40).

The most serious pathology that might trigger peri-implant edema and swelling after the use of orthopedic implants and prostheses is malignancy. Various animal studies have examined the biological effects of implants. Beryllium, cadmium, chromium, cobalt, iron, nickel, selenium, zinc, and titanium carcinogenicity studies have been conducted to demonstrate these effects [10-14]. However, some authors place the implant in accordance with the sarcoma found which can lead to the development of osteonecrosis [15]. A total of 31 cases of sarcoma associated with implants have been reported in the current literature. Such sarcomas may take various forms, the most common being malignant fibrous histiocytoma and osteosarcoma [3]. They behave aggressively and frequently metastasize. Sarcomas associated with implants may also develop in relation to the presence of metallosis and prosthetic debris. In 2001, Suzanne *et al*. [16] studied 12 patients who developed implant-associated sarcomas. Sarcomas were found in five patients after a total hip replacement, in four patients after the intramedullary nailing of fractures, in two patients after a staple application for osteotomy fixation, and in one patient after a plate fixation for a femoral fracture [3]. A stainless steel plate had been used to treat our patient. Similar reactions may thus be expected in other patients treated with stainless steel implants. However, such reactions should be differentiated from malignancies.In our case, there was no evidence of intra-operative metallosis. The dimensions of the removed mass approached 15×12×6cm. A pathologic examination revealed the cystic structures detected to have a wall thickness approaching 1mm. The largest of the cysts was 2cm in diameter, with the smallest being 2mm in diameter. The small cystic structures that were extruded from the mass were 3 to 4mm thick, numerous, dirty white in color, smooth-surfaced, rubber-like, and flat. Paraffin sections were stained with hematoxylin and eosin, and acellular, fibro-hyalinized cystic structures were observed (Figures 3 and 4). No protoscoleces were detected. Evaluation of the surrounding soft tissue revealed lymphocytes, polymorphonuclear leukocytes, and inflammatory cell infiltrations comprising histiocytes. The slices exhibited minimal cell wall structural features, mitosis, and/or necrosis. Atypical cells were not considered to indicate malignancy because of their rarity.

Although infection is generally the first possibility that should be considered in patients with pain and swelling in the vicinity of a prior implant, malignancies, hydatid cysts, and (finally) foreign body reactions should be kept in mind as differential diagnoses. No detailed investigation, such as magnetic resonance imaging, had previously been performed in our patient. Punctures were performed twice before the existence of greater trochanter bursitis was assumed, but the specimens were not sent for histopathological examination. This constitutes improper management of our patient. Insufficient pre-operative workup in such a case can result in an erroneous diagnosis, such as a hydatid cyst or a sarcoma. Therefore, we had planned to perform a wide resection following a possible diagnosis of malignancy or a hydatid cyst.

## Conclusions

In conclusion, the current literature does not report on any case exhibiting similar clinical and pathological findings to the best of our knowledge. A soft-tissue reaction around a previously implanted plate should be managed carefully. Our case is important because, despite the similarity between a macroscopic hydatid cyst and a sarcoma, they are only microscopically distinguishable from a soft-tissue reaction.

## Consent

Written informed consent was obtained from the patient for publication of this manuscript and any accompanying images. A copy of the written consent is available for review by the Editor-in-Chief of this journal.

## Abbreviations

CRP: C-reactive protein; CT: Computerized tomography.

## Competing interests

The authors declare that they have no competing interests.

## Authors’ contributions

AS, KB, SS and HHC contributed to the conception and design of the study, carried out the literature research, manuscript preparation and manuscript review. SO performed the histological examination of the mass, and was a contributor in writing the manuscript. MSS was involved with the case and writing of the manuscript, general management of the patient and revised the manuscript for important intellectual content. All authors read and approved the final manuscript.
